# Metabolic dysfunction-associated steatotic liver disease and new onset diabetes mellitus after liver transplantation

**DOI:** 10.1016/j.clinsp.2025.100806

**Published:** 2025-10-22

**Authors:** Marcelo Arouca Araujo, Mateus Jorge Nardelli, Rafael Pereira Freitas Mendes, Amanda Cássia da Cruz Cardoso, Guilherme Grossi Lopes Cançado, Henrique Drumond Braga, Livia Manussakis Vaz Ferreira, Luis Henrique de Oliveira Moreira, Letícia Chaves Victor da Silva, Anderson Antônio de Faria, Claudia Alves Couto, Luciana Costa Faria

**Affiliations:** a Serviço de Diagnóstico por Imagem do Hospital das Clínicas da Universidade Federal de Minas Gerais/EBSERH, Belo Horizonte, MG, Brazil; b Programa de Pós-Graduação em Ciências Aplicadas à Saúde do Adulto da Universidade Federal de Minas Gerais, Belo Horizonte, MG, Brazil; c Instituto Alfa de Gastroenterologia do Hospital das Clínicas da Universidade Federal de Minas Gerais/EBSERH, Belo Horizonte, MG, Brazil; d Faculdade de Medicina da Universidade Federal de Minas Gerais, Belo Horizonte, MG, Brazil

**Keywords:** Liver Transplantation, Nonalcoholic Fatty Liver Disease, Metabolic Dysfunction-Associated Steatotic Liver Disease, Fatty Liver, Post-Transplant Diabetes, Insulin Resistance, Metabolic Syndrome, Ultrasound, Hepatic elastography

## Abstract

**Introduction and objectives:**

After Liver Transplantation (LT), patients often gain weight and develop metabolic comorbidities. This study aimed to assess the prevalence and severity of Metabolic Dysfunction-Associated Steatotic Liver Disease (MASLD) after LT and the incidence of New-Onset Diabetes After LT (NODALT).

**Methods:**

Post-LT patients were recruited from an outpatient clinic. Hepatic ultrasound and 2D shear-wave elastography were performed.

**Results:**

A total of 142 patients were included, 63 % male with median age 60 (Interquartile Range [IQR] 47‒68 years). Median follow-up time post-LT was 137 (IQR 77‒205 months). MASLD was diagnosed in 26 %. Patients with MASLD exhibited a higher prevalence of central obesity (92 % vs. 68 %, *p* = 0.006), diabetes (65 % vs. 38 %, *p* = 0.005), hypertriglyceridemia (51 % vs. 26 %, *p* = 0.008), and metabolic syndrome (69 % vs. 41 %, *p* = 0.006) compared to those without MASLD. MASLD was independently associated with hypertriglyceridemia (Odds Ratio [OR = 2.80], 95 % Confidence Interval [95 % CI 1.22‒6.43], *p* = 0.015) and post-LT diabetes (OR = 2.65, 95 % CI 1.15‒6.10, *p* = 0.022). NODALT occurred in 36 % and was independently associated with older age (OR = 1.05, 95 % CI 1.01‒1.08, *p* = 0.01), time from LT (OR = 1.01, 95 % CI 1.003‒1.016, *p* = 0.006), body mass index (OR = 1.12, 95 % CI 1.02‒1.22, *p* = 0.02) and MASLD (OR = 2.99, 95 % CI 1.13‒7.92, *p* = 0.028). Advanced fibrosis occurred in 9 % of MASLD patients, which was similar from patients without MASLD.

**Conclusion:**

After LT, the prevalence of MASLD was 26 % ant the incidence of NODALT was 36 %. MASLD was associated with diabetes and hypertriglyceridemia, while NODALT was linked to older age, longer follow-up post-LT and MASLD. These findings emphasize the importance of preventing post-LT metabolic complications.

## Highlights

• After Liver Transplantation (LT), weight gain, MASLD and NODALT were frequent.
• MASLD was associated with diabetes and hypertriglyceridemia post-LT.
• NODALT was associated with older age, longer follow-up, body mass index and MASLD.
• It is crucial to prevent post-LT metabolic complications.

## Introduction

After Liver Transplantation (LT), patients often gain weight and develop Metabolic Syndrome (MS) and its components: obesity, Insulin Resistance (IR), arterial hypertension and dyslipidemia.^1^ Several factors contribute to this, such as the reversal of cirrhosis, increased appetite, and the use of immunosuppressive drugs.^2^^,^^3^ Metabolic Dysfunction-Associated Steatotic Liver Disease (MASLD) is considered the hepatic manifestation of MS and both the recurrent and *de novo* forms are common after LT.^4^ Previous studies have shown rates between 18 %‒40 % of *de novo* MASLD based on histological criteria.^4^, ^5^, ^6^, ^7^, ^8^ On the other hand, reported frequencies of recurrent MASLD have varied widely, ranging from 30 %‒100 %.^4^^,^^9^ New-Onset Diabetes Mellitus (DM) After LT (NODALT) is also a common complication,^10^ diagnosed in 15 %‒45 % of patients^11^ and associated with worst prognosis.^12^^,^^13^

Because of the prolonged patient survival after LT in recent years, the prevalence of MASLD and NODALT is probably increasing and so the related comorbidities, which is relevant due to the risk of higher cardiovascular death related to those conditions.^14^, ^15^, ^16^ In fact, NODALT is also associated with renal impairment^17^ and lower patient and graft survival.^18^

Brazil has experienced marked increases in overweight and obesity over the last few decades,^19^^,^^20^ reason why the prevalence of MASLD in the general adult population is higher than 30 %.^21^ The country has a highly admixed population, with varying proportions of Native American, African, and European genetic ancestry^22^ and performs over 1700 LT annually.^23^ However, there is limited data available on MASLD post-LT and NODALT in the Brazilian population. The aims of this study were to investigate the prevalence and severity of MASLD, the incidence of NODALT and the prevalence of MS and its components in a Brazilian cohort of liver transplant recipients.

## Methods

### Study population and design

Cross-sectional study enrolled LT recipients aged 18-years or older who attended the Transplant Outpatient Clinic at the Hospital das Clínicas da Universidade Federal de Minas Gerais/EBSERH in Belo Horizonte, Brazil, between November 2021 and November 2022.

The exclusion criteria were <12-months of follow-up after LT, alcohol intake greater than 20 g/day or 140 g/week for women and 30 g/day or 210 g/week for men, active HIV infection, recurrence of Hepatocellular Carcinoma (HCC), and diagnosis of neoplasm other than skin in the past two years. The study was approved by the Ethics Committee of Human Research from Universidade Federal de Minas Gerais (CAAE 47,621,821.1.0000.5149) and participants were included after signing the informed consent form.

### Anthropometric, clinical and laboratory data

Anthropometric measurements included height, weight and Waist Circumference (WC). Weight was collected before LT, in the first outpatient visit after LT, one and three-years post-LT and at the time of inclusion in this study. Body Mass Index (BMI) was calculated using the weight/height^2^ formula. Obesity was defined as a BMI ≥ 30 kg/m^2^.^24^ WC was measured at the midpoint between the last costal arch and the iliac crest, using a non-extensible measuring tape. Central obesity was defined as WC ≥ 80 cm for women and WC ≥ 90 cm for men.^25^

Clinical and epidemiological data, such as sex, age, date of LT, indication for LT, as well as pre- and post-transplant comorbidities and immunosuppression regimens, were gathered from patients' medical records and through interviews. Laboratory tests were recorded if performed within a maximum interval of three months before or after the inclusion in the study. These tests included fasting blood glucose and glycohemoglobin levels, creatinine, total cholesterol, Low-Density Lipoprotein Cholesterol (LDL-c), High-Density Lipoprotein Cholesterol (HDL-c), triglycerides, serum albumin, bilirubin, platelet count, Aspartate Aminotransferase (AST), and Alanine Aminotransferase (ALT).

The criteria for the diagnosis of MASLD were the detection of liver steatosis by ultrasound, along with the presence of at least one out of five cardiometabolic risk factors: 1) BMI ≥ 25 kg/m^2^ or WC ≥ 94 cm (male) or 80 cm (female); 2) Fasting serum glucose ≥ 100 mg/dL or HbA1c ≥ 5.7 % or documented diabetes diagnosis or treatment; 3) Blood pressure ≥ 130/85 mmHg or specific antihypertensive drug treatment; 4) Plasma triglycerides ≥ 150 mg/dL or lipid lowering treatment; 5) Plasma HDL-*c* ≤ 40 mg/dL (male) or ≤ 50 mg/dL (female) or lipid lowering treatment; and the absence of other causes of liver steatosis.^26^

Diabetes was defined according to the American Diabetes Association criteria^27^ and, for the diagnosis of NODALT, patients who already had diabetes prior to LT were excluded. MS was defined according to the International Diabetes Federation.^25^ Dyslipidemia was defined as LDL-*c* ≥ 130 mg/dL or triglycerides ≥ 150 mg/dL or HDL-*c* < 40 mg/dL for men or < 50 mg/dL for women or the use of lipid-lowering drugs. Hypertension was considered if values were equal to or exceeded 140 mmHg for systolic and 90 mmHg for diastolic blood pressure, or if patients were taking medications to manage hypertension.

### Liver ultrasound and elastography

All the participants underwent hepatic ultrasound and elastography using the 2D Shear Wave Elastography (2D-SWE) technique, performed by a single experienced radiologist, using an ultrasound machine with a C1–6 convex transducer (GE Logiq S8) to identify the presence of hepatic steatosis and determine liver stiffness.

The assessment of liver stiffness by 2D-SWE followed the protocol recommended by the Society of Radiologists in Ultrasound.^28^ Patients were evaluated after fasting for at least four hours, in dorsal decubitus or slightly left lateral decubitus (30°), with the right arm up. Measurements were taken in the right lobe with the transducer positioned in the intercostal space, during apnea. Twelve measurements were taken for each patient, with the color box measuring 2 × 3 cm, positioned at least 2 cm below the liver capsule, and the perivascular region was avoided. For each acquisition, a Region Of Interest (ROI) was selected after stabilization of the rigidity color map. For each patient, liver stiffness was defined as the median of twelve measures considered adequate, in m/s, when the Interquartile Range/Median Ratio (IQR/M) was less than or equal to 15 %. To quantify liver stiffness, the recommendations of the device manual (Logiq S8 R3 from GE) were followed and the results were classified as follows: a) Absent fibrosis: < 1.47 m/s; b) Mild fibrosis: 1.47–1.48 m/s; c) Mild to moderate fibrosis: 1.48–1.64 m/s; d) Moderate to severe fibrosis: 1.64–1.76 m/s; e) Cirrhosis: > 1.76 m/s, corresponding to METAVIR classification F0, F1, F2, F3 and F4, respectively.

### Statistical analysis

Statistical analyses were performed using the Statistical Package for the Social Sciences (SPSS) for Windows, version 22.0 (SPSS Inc., Chicago, IL, USA). Shapiro-Wilk test was used to test the normality of the data. Continuous variables were presented as means and Standard Deviations (SD) or medians and Interquartile Ranges (IQR), while dichotomous variables as absolute numbers and percentages. Group differences for continuous variables were analyzed using Student’s *t*-test or the Mann-Whitney *U* test, as appropriate. Comparison of categorical variables was performed using the chi-square test or Fisher’s exact test, as appropriate. The prevalence of hypertension, diabetes and obesity before and after LT were compared using the McNemar test. Multivariate logistic regression was used to evaluate the variables independently associated with MASLD and NODALT. In the multivariate models, variables with clinical relevance and with *p* < 0.20 in the univariate analysis were included if they did not have multicollinearity. Statistical significance was set at two-sided p values <0.05.

## Results

### Study group

From the initial 226 recruited patients, 35 refused to participate the study and 45 did not attend the scheduled examination date. A total of 146 patients underwent radiological examination, but two patients were excluded due to neoplasm diagnosis (non-Hodgkin’s lymphoma and mouth squamous cell carcinoma) and other two subjects were excluded because they had <12-months of follow-up after transplantation.

Final sample included 142 liver transplanted patients who met the inclusion criteria (62.7 % males). The median age was 60 (IQR 47–68 years) and the median follow-up time was 137 (IQR 77–205 months). Cohort characteristics are described in Table 1.

**Table 1:** Clinical, demographic, anthropometric and laboratory characteristics of liver transplanted patients with and without Metabolic Dysfunction-Associated Steatotic Liver Disease (MASLD).

Variables	Cohort (n = 142)	No‒MASLD (n = 105)	MASLD (n = 37)	p-value
Male sex	89 (62.7)	66 (62.9)	23 (62.2)	0.940^a^
Age (years)	60 (47‒68)	59 (46‒68)	60 (51‒66)	0.747^d^
Time since LT (months)	137 (77‒205)	142 (74‒210)	136 (77‒169)	0.450^d^
Liver disease etiology				
Alcohol related liver disease	30 (21.1)	23 (21.9)	7 (18.9)	0.753^b^
Autoimmune liver disease	29 (20.4)	23 (21.9)	6 (16.2)	
Viral hepatitis	40 (28.1)	30 (28.6)	10 (27.0)	
Cryptogenic cirrhosis	20 (14.1)	14 (13.3)	6 (16.2)	
MASLD	6 (4.2)	3 (2.9)	3 (8.1)	
Other causes	17 (12.0)	12 (11.4)	5 (13.5)	
Hepatocellular carcinoma at LT	35 (24.6)	26 (24.8)	9 (24.3)	0.958^a^
Comorbidities before LT				
Diabetes mellitus	21 (14.8)	13 (12.4)	8 (21.6)	0.173^a^
Arterial hypertension	20/134 (14.9)	11/98 (11.2)	9/36 (25.0)	0.047^a^
Comorbidities after LT				
Diabetes mellitus	64 (45.1)	40 (38.1)	24 (64.9)	0.005^a^
Arterial hypertension	78 (54.9)	57 (54,3)	21 (56.8)	0.795^a^
Dyslipidemia	77/127 (60.6)	53/92 (57.6)	24/35 (68.6)	0.259^a^
Hypertriglyceridemia	42/126 (33.3)	24/91 (26.4)	18/35 (51.4)	0.008^a^
High LDL-c	16/125 (12.8)	12/90 (13.3)	4/35 (11.4)	>0.999^b^
Low HDL-c	44/125 (35.2)	33/90 (36.7)	11/35 (31.4)	0.582^a^
Metabolic syndrome	65/134 (48.5)	41/99 (41.4)	24/35 (68.6)	0.006^a^
Current immunosuppression				
Tacrolimus	131 (92.3 %)	97 (92.4 %)	34 (91.9 %)	>0.999^b^
Prednisone	26 (18.3 %)	21 (20 %)	5 (13.5 %)	0.380^b^
Anthropometric data				
BMI before LT (kg/m^2^)	26.3 (23.5‒30.5)	26.1 (23.3‒29.4)	27.2 (24.7‒32.4)	0.084^d^
BMI at first visit after LT (kg/m^2^)	22.7 (20.8‒27.3)	22.4 (20.7‒25.6)	24.7 (21.0‒29.2)	0.065^d^
BMI at the time of US (kg/m^2^)	26.7 (23.4‒30.0)	25.8 (22.4‒29.6)	28.2 (24.8‒31.3)	0.008^d^
∆ Weight 1 yr. after LT ( %)	8.9 ± 13.6	8.2 ± 13.2	10.4 ± 14.7	0.438^c^
∆ Weight since LT ( %)	12.6 (3.9‒25.2)	11.5 (3.9‒22.1)	15.9 (7.8‒34.7)	0.209^d^
Obesity	36 (25.4)	23 (21.9)	13 (35.1)	0.127^a^
Central obesity	104/140 (74.3)	71/104 (68.3)	33/36 (91.7)	0.006^a^
Radiologic exams				
Fatty liver	37 (26.1)	–	–	–
Liver stiffness (m/s)	1.38 (1.28‒1.47)	1.38 (1.28‒1.47)	1.38 (1.31‒1.46)	0.898^d^
Advanced fibrosis	16/138 (11.6)	13/104 (12.5)	3/34 (8.8)	0.561^b^
Laboratory tests				
Creatinine clearance (mL/min)	74 (55‒96)	72 (53‒95)	84 (62‒97)	0.191^d^
Fasting glucose (mg/dL)	100 (87‒126)	96 (86‒114)	114 (94‒149)	0.002^d^
Glycohemoglobin ( %)	5.7 (5.2‒6.5)	5.6 (5.2‒6.3)	5.9 (5.6‒7.1)	0.007^d^
Triglycerides (mg/dL)	124 (92‒158)	119 (85‒142)	145 (104‒205)	0.007^d^
Total cholesterol (mg/dL)	171 (143‒198)	163 (142‒198)	185 (151‒196)	0.289^d^
HDL-c (mg/dL)	48 (39‒60)	47 (39‒61)	50 (38‒59)	0.998^d^
LDL-c (mg/dL)	95 ± 30	96 ± 31	93 ± 29	0.713^c^
AST (U/L)	28 (22‒36)	28 (22‒35)	30 (22‒41)	0.334^d^
ALT (U/L)	24 (17‒32)	22 (17‒29)	29 (21‒50)	0.019^d^
Total bilirubin (mg/dL)	0.7 (0.5‒0.9)	0.7 (0.6‒0.9)	0.6 (0.5‒0.9)	0.127^d^
Serum albumin (g/dL)	4.5 ± 0.4	4.5 ± 0.4	4.6 ± 0.3	0.235^c^
Total platelets (10^3^/mm^3^)	171 (134‒212)	170 (131‒207)	173 (140‒224)	0.411^d^

Data are presented as: absolute number/available data (percentage), mean ± standard deviation or median (interquartile interval). MASLD, Metabolic Dysfunction-Associated Steatotic Liver Disease; ALT, Alanine Aminotransferase; AST, Aspartate Aminotransferase; HDL-c, High-Density Lipoprotein Cholesterol; BMI, Body Mass Index; US, Ultrasound; LDL-c, Low-Density Lipoprotein cholesterol; LT, Liver Transplantation.

a Chi-square test.

b Fisher’s exact test.

c Student t-test.

d Mann-Whitney test.

The main indications for LT were viral hepatitis (28.1 %) and alcohol-related cirrhosis (21.1 %), followed by autoimmune liver diseases (20.4 %). Thirty-five patients (24.6 %) had HCC at the time of LT. At the time of evaluation, study participants were following various immunosuppressive regimens: tacrolimus (53 %); tacrolimus and mycophenolate mofetil (CellCept) (MMF) (20 %); tacrolimus, MMF and prednisone (10 %); tacrolimus and prednisone (5.6 %); MMF and everolimus (2.1 %); and cyclosporine (Cyclosporine capsules) (2.1 %). The remaining patients were using different combinations of immunosuppressants. In total, 131 patients (92 %) were taking tacrolimus, 47 (33.1 %) MMF, 26 (18 %) prednisone, 7 (4.9 %) were on everolimus, and 5 (3.5 %) were using cyclosporine (Cyclosporine capsules).

### Prevalence and risk factors for MASLD in LT patients

MASLD was diagnosed in 37 patients (26.1 %). Recurrent MASLD was identified in three out of six patients (50 %) who underwent LT for Metabolic Dysfunction-Associated Steatohepatitis (MASH). De novo MASLD was observed in 34 out of 136 (25 %) participants with other indications for LT. Comparison between patients with and without MASLD post-LT is shown in Table 1. Features associated with MASLD post-LT were pre-LT hypertension, post-LT diabetes, hypertriglyceridemia, MS, BMI, central obesity, fasting glucose, glycohemoglobin, triglycerides and ALT levels. In a multivariate analysis including the variables post-LT diabetes, hypertriglyceridemia and central obesity, those independently associated with MASLD were post-LT hypertriglyceridemia (OR = 2.80, 95 % CI 1.22‒6.43, *p* = 0.015) and diabetes (OR = 2.65, 95 % CI 1.15‒6.10, *p* = 0.022).

### Hepatic fibrosis by 2D-SWE

Characteristics of MASLD patients with 2D-SWE velocities ≥ 1.47 m/s are described in Table 2. All of them had diabetes and central obesity and 6 out of 7 (85.7 %) presented MS. The prevalence of advanced hepatic fibrosis (F3 and F4) in patients with MASLD was 8.8 %, which was not significantly different from patients without MASLD.

**Table 2:** Characteristics of patients with MASLD and fibrosis after LT by hepatic ultrasound and 2D-SWE.

	Patients with MASLD and fibrosis						
	1	2	3	4	5	6	7
Age (years)	43	65	56	60	68	69	51
Sex	F	F	M	M	M	M	M
Etiology of cirrhosis before LT	Autoimmune	Virus C	Cryptogenic	Hemochromatosis	Ethanolic	Ethanolic	Ethanolic
Follow up post-LT (months)	305	138	122	39	158	107	92
BMI (Kg/m²)	30.2	24.6	31.3	30.5	24.8	32.2	49.8
WC (cm)	102	91	110	102	103	112	134
Diabetes	Yes	Yes	Yes	Yes	Yes	Yes	Yes
Hypertension	No	Yes	Yes	No	Yes	Yes	No
Metabolic syndrome	Yes	Yes	Yes	Yes	Yes	Yes	No
Fasting glucose (mg/dL)	115	150	140	127	143	113	174
Triglycerides (mg/dL)	122	116	171	213	–	132	94
LDL-c (mg/dL)	74.2	112.4	94.0	96.6	–	49.9	47.5
HDL-c (mg/dL)	86	59	41	48	–	41	80
AST (U/L)	51	41	27	22	38	50	36
ALT (U/L)	51	36	44	23	56	40	29
GGT (U/L)	84	66	189	72	32	163	26
2D-SWE (METAVIR)	F1	F4	F2	F1	F2	F3	F2
2D-SWE (m/s)	1.47	2.02	1.49	1.47	1.50	1.74	1.53
Liver biopsy	AIH recurrence + de novo MASH, Fibrosis F3 (4.5y before 2D-SWE)	‒	De novo MASH (3.5y before 2D-SWE)	Mild perisinusoidal fibrosis and macrovesicular steatosis in zone 3 (2 m before 2D-SWE)	‒	De novo MASH, perisinusoidal fibrosis (2y before 2D-SWE)	–

MASLD, Metabolic Dysfunction-Associated Steatotic Liver Disease; LT, Liver Transplantation; 2D-SWE, 2D Shear Wave Elastography; M, Male; F, Female; BMI, Body Mass Index; WC, Waist Circumference; LDL-c, Low Density Lipoprotein Cholesterol; HDL-c, High Density Lipoprotein cholesterol; AST, Aspartate Aminotransferase; ALT, Alanine Aminotransferase; GGT, Gamma Glutamyl Transferase; AIH, Autoimmune Hepatitis; MASH, Metabolic Dysfunction-Associated Steatohepatitis; y, years; m, months.

### Incidence of NODALT and associated features

Prior to LT, 21 patients (14.8 %) had diabetes. After LT, NODALT occurred in 43 out of 121 patients (35.5 %), resulting in a total of 64 patients (45.1 %) with diabetes at the time of this study. Comparison between patients with and without NODALT are presented in Table 3. NODALT was associated with higher age, longer follow-up time since LT, BMI before LT, at the first outpatient appointment after LT, at the time of US, obesity, central obesity and presence of MASLD. In a multivariate analysis including the variables age, time elapsed since LT, BMI and MASLD, all of them were independently associated with NODALT: age (OR = 1.046, 95 % CI 1.010‒1.084, *p* = 0.011), time from LT (OR = 1.009, 95 % CI 1.003‒1.016, *p* = 0.006), BMI (OR = 1.116, 95 % CI 1.019‒1.223, *p* = 0.019) and MASLD (OR = 2.985, 95 % CI 1.125‒7.922, *p* = 0.028).

**Table 3:** Comparison between patients with and without diagnosis of New Onset Diabetes Mellitus After Liver Transplantation (NODALT).

Variables	No‒NODALT (n = 78)	NODALT (n = 43)	p-value
Male sex	49 (62.8)	27 (62.8)	0.997^a^
Age (years)	54 (40‒64)	62 (56‒68)	0.003^d^
Time since LT (months)	131 (67‒183)	182 (124‒218)	0.006^d^
Liver disease etiology			
Alcoholic liver disease	13 (16.7)	11 (25.6)	0.234^a^
Autoimmune liver disease	23 (29.5)	5 (11.6)	
Viral hepatitis	19 (24.4)	12 (27.9)	
Cryptogenic cirrhosis	11 (14.1)	8 (18.6)	
MASLD	1 (1.3)	2 (4.7)	
Other causes	11 (14.1)	5 (11.6)	
Hepatocellular carcinoma at LT	17 (21.8)	9 (20.9)	0.912^a^
Comorbidities before LT			
Arterial hypertension	8/74 (10.8)	6/39 (15.4)	0.483^a^
Comorbidities after LT			
Arterial hypertension	40 (51.3)	25 (58.1)	0.469^a^
Dyslipidemia	39/66 (59.1)	24/41 (58.5)	0.955^a^
Hypertriglyceridemia	19/66 (28.8)	16/41 (39.0)	0.273^a^
High LDL-c	11/65 (16.9)	4/41 (9.8)	0.303^a^
Low HDL-c	25/65 (38.5)	10/41 (24.4)	0.134^a^
Current immunosuppression			
Tacrolimus	74 (94.9 %)	36 (83.7 %)	0.052
Prednisone	18 (23.1 %)	7 (16.3 %)	0.377
Anthropometric data			
BMI before LT (kg/m^2^)	25.5 (23.4‒28.7)	27.8 (24.6‒32.6)	0.020^d^
BMI at first visit after LT (kg/m^2^)	22.2 (20.6‒24.6)	25.0 (22.0‒29.0)	0.015^d^
BMI at the time of inclusion (kg/m^2^)	25.2 (22.7‒28.3)	27.4 (24.4‒31.7)	0.020^d^
∆ Weight 1 yr. after LT ( %)	7.8 ± 13.0	8.9 ± 14.6	0.721^c^
∆ Weight since LT ( %)	12.7 (3.4‒27.6)	11.8 (5.2‒21.7)	0.931^d^
Obesity	13 (16.7)	16 (37.2)	0.011^a^
Central obesity	52 (66.7)	36/42 (85.7)	0.024^a^
Radiologic exams			
Fatty liver	13 (16.7)	16 (37.2)	0.011^a^
Liver stiffness (m/s)	1.40 (1.30‒1.47)	1.35 (1.28‒1.46)	0.306^d^
Advanced fibrosis	9/77 (11.7)	6/41 (14.6)	0.647^b^
Laboratory exams			
Creatinine clearance (mL/min)	77 (60‒101)	74 (53‒96)	0.437^d^
Triglycerides (mg/dL)	118 (88‒146)	128 (92‒171)	0.146^d^
Total cholesterol (mg/dL)	174 ± 36	175 ± 35	0.820^c^
HDL-c (mg/dL)	46 (39‒59)	50 (42‒63)	0.190^d^
LDL-c (mg/dL)	99 ± 30	92 ± 32	0.245^c^
AST (U/L)	28 (22‒35)	32 (25‒39)	0.134^d^
ALT (U/L)	23 (17‒29)	25 (18‒46)	0.206^d^
Total bilirubin (mg/dL)	0.7 (0.5‒0.9)	0.6 (0.5‒0.9)	0.811^d^
Serum albumin (g/dL)	4.5 (4.2‒4.7)	4.6 (4.3‒4.8)	0.408^d^
Total platelets (10^3^/mm^3^)	176 (136‒211)	161 (130‒210)	0.655^d^

Data are presented as: absolute number/available data (percentage), mean ± standard deviation or median (interquartile range). MASLD, Metabolic Dysfunction-Associated Steatotic Liver Disease; NODALT, New Onset Diabetes Mellitus After Liver Transplantation; ALT, Alanine Aminotransferase; AST, Aspartate Aminotransferase; HDL-c, High-Density Lipoprotein cholesterol; LDL-c, Low-Density Lipoprotein cholesterol; LT, Liver Transplantation.

a Chi-Square test.

b Fisher’s exact test.

c Student t-test.

d Mann-Whitney test.

### Prevalence of hypertension, obesity, dyslipidemia and MS

The prevalence of hypertension and obesity at the time of this study was significantly higher than those of hypertension before LT and of obesity at the first outpatient appointment after LT (54.9 % vs. 14.9 %, *p* < 0.01 for hypertension and 25.4 % vs. 11.4 %, *p* < 0.01 for obesity). The prevalence of dyslipidemia was 60.6 % and of MS, 48.5 %.

### Weight gain and association with comorbidities

**Fig. 1:**
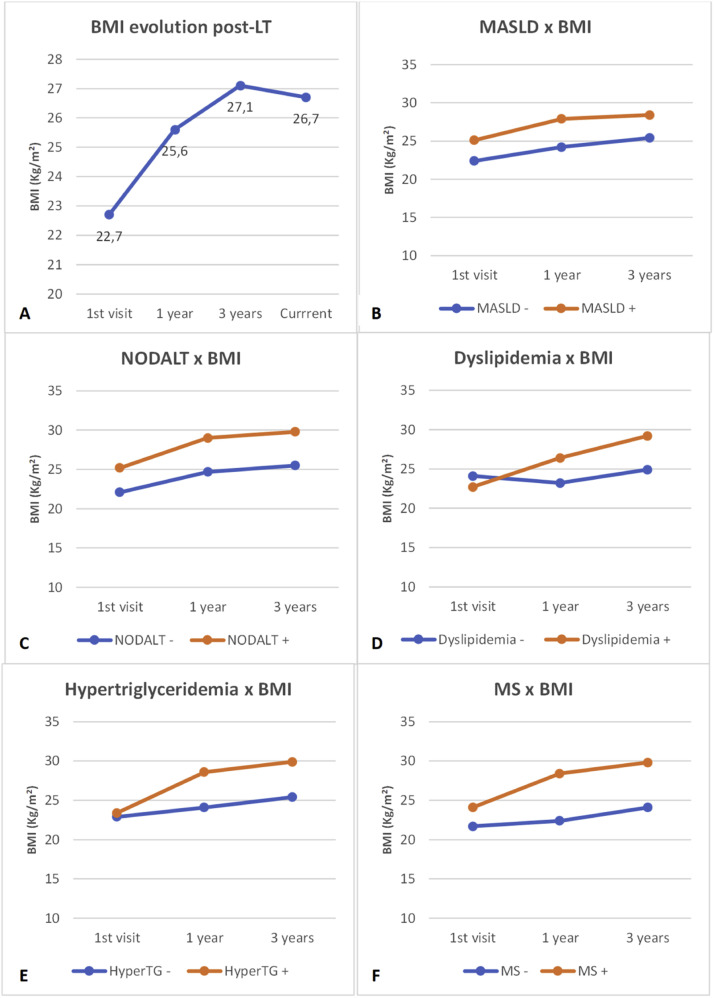
(A) BMI evolution at the evaluation period. (B, C, D, e F) Temporal variation in body mass index post-liver transplantation in patients with and without MASLD, NODALT, dyslipidemia, hypertriglyceridemia and metabolic syndrome. BMI, Body Mass Index; LT, Liver Transplantation; MASLD, Metabolic Dysfunction-Associated Stetatotic Liver Disease; NODALT, New Onset Diabetes Mellitus After Liver Transplantation; HyperTG, Hypertriglyceridemia; MS, Metabolic Syndrome.

Patients presented progressive weight gain after LT with an average gain of 8.9 ± 11.2 kg since the first outpatient appointment post-LT. The weight gain was more pronounced in the first three years after LT, after which it tended to stabilize (Fig. 1A). Patients with MASLD, NODALT, dyslipidemia, hypertriglyceridemia and MS had a significantly higher BMI one and three years after LT, compared to patients without these comorbidities. No differences were observed in the proportion of BMI increase during the first year and during the first three years post-LT between patients with and without MASLD and NODALT. In contrast, patients with dyslipidemia, hypertriglyceridemia, and MS displayed a greater increase in BMI during the first year and during the first three years after LT, in comparison to those without these metabolic comorbidities (Table 4 and Figs. 1B‒F).

**Table 4:** Comparisons of body mass index variations in relation to the first post-liver transplantation outpatient appointment at one and three years after transplantation in patients with and without MASLD, NODALT, dyslipidemia, hypertriglyceridemia and metabolic syndrome.

		1st outpatient appointment after LT	1-year post-LT		3-years post-LT	
		BMI (kg/m^2^)	BMI (kg/m^2^)	∆ Post-LT	BMI (kg/m^2^)	∆ Post-LT
MASLD	No	22.4	24.2	9 %	25.4	13 %
		(20.3–25.8)	(21.2–29.3)	(−3 – 15)	(22.1–29.9)	(4 – 23)
	Yes	25.1	27.9	7 %	28.4	16 %
		(21.1–29.3)	(24.2–30.7)	(−1 – 21)	(26.4–31.7)	(5 – 32)
	p-value		0.002	0.514	0.002	0.361
NODALT	No	22.1	24.7	8 %	25.5	13 %
		(19.9–26.1)	(21.3–27.4)	(−2 – 17)	(23.1–29.4)	(5 – 28)
	Yes	25.2	29.0	8 %	29.8	15 %
		(22.1–29.5)	(23.6–31.0)	(−2 – 15)	(24.6–33.9)	(4 – 25)
	p-value		0.003	0.888	0.006	0.854
Dyslipidemia	No	24.1	23.2	0 %	24.9	6 %
		(21.0–28.0)	(21.5–27.4)	(−11 – 10)	(22.3–27.7)	(0 – 17)
	Yes	22.7	26.4	14 %	29.2	18 %
		(20.9–27.2)	(24.1–20.0)	(5 – 20)	(25.3–31.7)	(9 – 33)
	p-value		0.018	<0.001	0.003	<0.001
Hyper trygliceridemia	No	22.9	24.1	6 %	25.4	12 %
		(20.6–27.3)	(21.4–27.2)	(−4 – 14)	(22.9–29.5)	(3 – 22)
	Yes	23.4	28.6	17 %	29.9	20 %
		(21.4–28.0)	(26.4–31.2)	(5 – 30)	(28.1–31.9)	(9 – 35)
	p-value		<0.001	<0.001	<0.001	0.018
Metabolic syndrome	No	21.7	22.4	4 %	24.1	7 %
		(19.5–24.9)	(21.5–28.0)	(−4 – 14)	(21.0–26.3)	(3 – 19)
	Yes	24.1	28.4	14 %	29.8	20 %
		(21.5–28.0)	(25.3–31.0)	(6 – 21)	(27.3–32.0)	(11 – 33)
	p-value		<0.001	<0.001	<0.001	0.001

Data are expressed as median (interquartile range). Mann-Whitney test was used to compare BMI variation in each time point compared with the BMI at first visit after LT inside each subgroup. BMI, Body Mass Index; NODALT, New Onset Diabetes mellitus After Liver Transplantation; MASLD, Metabolic Dysfunction-Associated Steatotic Liver Disease; LT, Liver Transplantation; ∆ Post-LT, difference between BMI in each time point and BMI at the first visit after LT ÷ BMI at the first visit after LT.

## Discussion

In this study, MASLD was identified in 26.1 % patients after LT, with *de novo* MASLD observed in 25 % of the patients. From patients with previous diagnosis of MASLD, 50 % recurred after LT. MASLD diagnosis was independently associated with post-LT diabetes and hypertriglyceridemia. It seems that MASLD did not increase the risk for advanced fibrosis. NODALT occurred in 36 % of patients, and it was independently associated with higher age, longer follow-up after LT, higher BMI and presence of MASLD. Patients with MASLD and NODALT, compared to those without these conditions, had higher BMI before and in the first three years after LT, even though there was no difference in the BMI increase during that follow-up, showing that baseline BMI may be determinant to these metabolic complications after LT.

Our results are in agreement with previous studies that indicate an incidence of recurrent and *de novo* MASLD of 30 %‒100 % and 18 %‒40 %, respectively.^4^^–^^9^ In a meta-analysis enrolling 2166 subjects, the pooled prevalence of *de novo* MASLD was 26 % at a follow-up period of six months to 10-years.^8^ There is a wide variability in the recurrence or *de novo* rates depending on the diagnostic method that is used: liver tests, histology or imaging techniques. In the present study, abdominal US was used, which is known to have lower sensitivity than liver biopsy and Proton Density Fat Fraction Magnetic Resonance Imaging (PDFF-MRI) in cases of mild steatosis. Liver biopsy is an invasive method with associated risks and is not routinely performed after LT in our service and PDFF-MRI is not available. A previous Brazilian study, also using ultrasound as a diagnostic tool, showed a prevalence of 43 % of fatty liver in patients after LT, being recurrent and *de novo* MASLD observed in 56 % and 43 % of the patients, respectively.^29^

The main risk factors for post-LT MASLD identified in previous studies are diabetes, hypertension, hyperlipidemia, obesity/weight gain, genetic polymorphism PNPLA3, graft steatosis, LT indication (i.e., MASH, HCV, alcoholic liver disease) and immunosuppression.^30^^,^^31^ In the present study, MASLD after LT was associated with hypertension before LT, post-LT diabetes, hypertriglyceridemia, central obesity and MS. The findings of this study are consistent with those of other studies, highlighting the significant role of post-transplant BMI as a crucial risk factor for MASLD.^4^ It's worth noting that, in contrast to previous research,^8^^,^^31^ the present study did not find an association between MASLD and any specific indication for LT. These variations might be attributed to differences in the study populations, patient demographics, and possibly other factors influencing the development of MASLD in post-transplant individuals. Additionally, donor characteristics, particularly hepatic steatosis and diabetes, have been showed to be associated with worse outcomes after LT.^32^ Some studies suggest that donor steatosis may be a risk for post-LT fatty liver, especially in living donors,^33^ but this finding is not consistent in similar studies, with recipient metabolic factors being more associated with post-LT MASLD.^34^^,^^35^ Absence of donor metabolic characteristics was a limitation of our study.

Among the 37 patients diagnosed with post-LT MASLD, seven exhibited some degree of fibrosis as identified by 2D-SWE. All seven of these patients had diabetes and central obesity, while six also had MS. In three of the patients with MASLD, advanced fibrosis (Grade 3 or 4) was detected, two of those identified through 2D-SWE and the other by liver biopsy. Interestingly, the prevalence of advanced fibrosis in patients with MASLD did not differ significantly from that observed in transplanted patients without MASLD. It's important to note that two of the three patients with advanced fibrosis also had other potential causes for hepatic fibrosis, i.e., autoimmune hepatitis and hepatitis C recurrence (which had already been successfully treated at the time of US assessment). Vallin et al. analyzed sequential liver biopsies from a cohort of 91 LT patients with MASLD and found that advanced fibrosis at five years post-LT was more frequent in recurrent (71.4 %) than in *de novo* MASLD (12.5 %).^4^^,^^36^ These findings were not reproduced in our study, maybe due to the small number of patients whose indication for transplantation was MASH. Other authors observed low rates of advanced fibrosis and MASLD-associated graft loss in patients transplanted for MASH and concluded that, although MASLD or MASH can recur, the clinical significance of disease recurrence for graft or patient survival may be small.^37^ It is important to emphasize that liver steatosis and fibrosis were identified by US and 2D-SWE in our study and by liver biopsy samples in most of other studies.^4^^,^^8^^,^^36^ US imaging does not reliably identify fat infiltration below 30 % of the parenchyma and the accuracy of shear wave elastography in the LT setting has not been established yet.

NODALT has been associated with elevated risk of cardiovascular disease, lower long-term patient and graft survival.^38^, ^39^, ^40^ In the present study, it was observed in 35.5% of the individuals. These findings are in line with previous studies that showed NODALT in 15%‒45%.^11^^,^^38^^,^^40^ In those reports, risk factors for NODALT were age,^38^^,^^40^ BMI,^38^, ^39^, ^40^ hepatitis C virus infection,^38^, ^39^, ^40^ male gender,^39^ pre-LT impaired fasting glucose,^39^ a family risk for diabetes,^39^ and tacrolimus-based immunosuppression.^38^^,^^39^ In our study, NODALT was associated with higher BMI and age, longer follow-up after LT, obesity and MASLD but not with hepatitis C infection or male gender. It is interesting to notice that NODALT was not associated with the proportion of weight gain, but with higher BMI before, at first visit after LT and at the time of the US. NODALT pathogenesis has not been fully elucidated yet but there is evidence of involvement of graft steatosis and genotype, recurrence of primary liver diseases, β-cells dysfunction induced by immunosuppressive drugs and cirrhosis, and gut microbiota dysbiosis.^41^

Most cirrhotic patients awaiting LT are malnourished and therefore some weight gain after LT seems to be considered appropriate.^42^ However, a significant number of patients experience excessive weight gain and develop overweight or obesity.^29^^,^^42^ The potential consequences include an increased risk of diabetes, hypertension, dyslipidemia, MS and associated complications, including cardiovascular disease. In this study, weight gain followed a progressive pattern in the first three years post-LT, with more pronounced gains during the first year and stabilization occurring after the third year. At the time of assessment (median follow-up of 137-months), the average weight gain was 8.9 ± 11.2 kg, and a considerable proportion of patients were overweight (59.9 %), including 25.4 % who were classified as obese. Mechanisms leading to excessive weight gain in this population are probably multifactorial, including the persistence of pre-transplant metabolic risk factors, lower energy expenditure following LT, adverse effects of immunosuppressive drugs, all of them contributing to the development of MS and MASLD after LT. In this sense, we observed that greater weight gain was associated with MS, hypertriglyceridemia and dyslipidemia, but not with MASLD and NODALT.

Taking into consideration epidemiologic and sociodemographic singularities of Latin America, our findings may contribute to the better understanding of metabolic dysfunction after LT. International data has recognized that metabolic dysfunction such as obesity, metabolic syndrome and diabetes are the main risk factors for post-LT steatosis and are associated with worse outcomes.^35^^,^^43^ The American continent has been showed to present higher prevalence of post-LT liver steatosis when compared to Europe or Asia,^35^ which may be due to regional factors such as nutritional habits, lifestyle and health assistance. Therefore, our findings highlight that strategies for preventing and treating obesity and diabetes in LT individuals are a key tool for better outcomes post-LT, especially if adapted for regional singularities.

This study has limitations. Firstly, the small sample size and the limited subgroups sizes restrict us to draw definite conclusions. Second, even though hepatic ultrasound is recommended by international guidelines as the initial imaging modality for detecting liver steatosis,^44^ its sensitivity significantly decreases for mild cases, potentially leading to underestimation of the true prevalence. Additionally, ultrasound is subject to inter-examiner variability. Although liver biopsy and magnetic resonance imaging remain as the most accurate methods for steatosis detection, they are tools that, in addition to being high cost, involve, in the first case, an invasive method with risk to the patient and, in the second, low availability.^44^ Furthermore, the cross-sectional design and diverse follow-up times are also limitation that require further prospective investigations with larger samples to validate our findings.

In conclusion, among individuals undergoing LT, the prevalence of MASLD was 26.1 %, and the incidence of NODALT was 35.5 %. MASLD was independently associated with post-LT diabetes and hypertriglyceridemia, while NODALT was independently associates with older age, longer follow-up time post-LT, obesity, and MASLD. Furthermore, there was a high prevalence of metabolic comorbidities after LT, as patients experienced significant weight gain after LT, particularly during the first year, with a subsequent deceleration in weight gain and stabilization after the third year. Therefore, it is essential to prioritize nutritional monitoring and encourage physical activity earlier in the post-LT follow-up to prevent metabolic complications after transplantation.

## Authors’ contributions

Conceptualization: MAA, CAC, LCF. Supervision: LCF. Data curation: MAA, RPFM, ACCC, HDB, LMVF, LHOM, LCVS, AAF, LCF. Investigation: MAA, LCF, RPFM, HDB, LMVF, LCVS. Formal analysis: MAA, MJN, LCF. Writing - original draft: MAA, MJN, LCF. Writing - review and editing: GGLC, AAF.

## Declaration of competing interest

The authors declare no conflicts of interest.
